# Nuclear translocation of triosephosphate isomerase 1 under aerobic glycolytic conditions is associated with HIV-1 transcription

**DOI:** 10.1016/j.jbc.2026.113148

**Published:** 2026-05-16

**Authors:** Towa Abe, Tae Yasutake, Satoshi Miura, Nobutoki Takamune, Shogo Misumi, Naoki Kishimoto

**Affiliations:** 1 Department of Environmental and Molecular Health Sciences, Faculty of Medical and Pharmaceutical Sciences, Kumamoto University, Chuo, Kumamoto, Japan; 2 Headquarters for Research and Development Strategy, Kumamoto University, Chuo, Kumamoto, Japan

**Keywords:** HIV-1, TPI1, aerobic glycolysis, viral production, nuclear translocation, HIV-1 transcription, metabolic reprogramming

## Abstract

The metabolic state of HIV-1-infected cells influences viral gene expression, with aerobic glycolytic conditions favoring HIV-1 transcription. Here, we demonstrate that under aerobic glycolytic conditions in HIV-1-infected cells, triosephosphate isomerase 1 (TPI1) undergoes nuclear translocation and promotes HIV-1 transcription. HIV-1-infected cells cultured in glucose-containing medium, which promotes aerobic glycolytic conditions, exhibited significantly higher levels of HIV-1 transcription than those cultured in galactose-containing medium, which favors oxidative phosphorylation. Under glucose conditions, TPI1 displayed a characteristic acidic isoform in HIV-1-infected cells. Overexpression of TPI1 increased this acidic isoform and enhanced HIV-1 transcription, whereas the S80A mutant failed to do so. These findings indicate that Ser^80^ plays a critical role in TPI1 nuclear localization and HIV-1 transcription. Consistently, nuclear TPI1 levels were markedly reduced under galactose conditions, in S80A-expressing cells, or upon TPI1 knockdown. Reduced nuclear TPI1 levels were associated with decreased H3K27 acetylation and diminished HIV-1 transcriptional activity. Moreover, TPI1 localization was unaffected by metabolic conditions in uninfected PBMCs but showed nuclear translocation under aerobic glycolytic conditions in HIV-1–infected PBMCs. Collectively, these findings suggest that, in HIV-1–infected cells, aerobic glycolytic conditions promote TPI1 nuclear translocation, thereby linking cellular metabolism to HIV-1 transcription.

Viruses depend on host cellular machinery for genome replication and gene expression. To replicate efficiently, viruses utilize host cell enzymes and transcriptional and translational machinery (1). Host cells also mount antiviral responses that restrict viral replication (2, 3). Understanding viral replication therefore requires defining how host cellular defense mechanisms influence viral gene expression. Viral infections are often associated with pronounced metabolic remodeling in host cells (4). Accordingly, it is important to define how metabolic state–responsive proteins contribute to viral gene regulation.

HIV-1 preferentially infects activated CD4^+^ T cells. Upon activation, CD4^+^ T cells reprogram their metabolism from oxidative phosphorylation (OXPHOS) to aerobic glycolysis, supporting proliferation and functional differentiation (5). A study of T cell activation showed that stimulation of lymphocytes with concanavalin A promotes intracellular glucose consumption as well as nucleic acid and protein synthesis (6). Metabolic engagements and reprogramming in immune and non-immune cells after viral recognition regulate the natural course of viral infection (7). Interestingly, HIV-1 infection in activated CD4^+^ T cells enhances the glycolytic flux to support viral production (8). We previously reported that a glucose-dependent metabolic state drives the production of highly infectious viruses (9). Together, these studies suggest that aerobic glycolysis facilitates HIV-1 replication. However, it remains unclear how the diverse protein modulation occurring under aerobic glycolytic conditions in CD4^+^ T cells impacts viral replication.

Glycolytic enzymes perform diverse functions beyond their canonical roles in metabolism. Proteins exhibiting multiple mechanistically distinct functions are referred to as “moonlighting proteins”, and glyceraldehyde 3-phosphate dehydrogenase (GAPDH) is widely recognized as one of them (10). Although no metabolic activity occurs within viral particles, we previously found that the three glycolytic enzymes, GAPDH, alpha-enolase, and pyruvate kinase muscle type 2, are incorporated into HIV-1 particles and modulate viral replication (11, 12, 13, 14). In addition, the incorporation of these proteins was inhibited under aerobic glycolytic conditions (9). These findings suggest a mechanistic link between glycolytic enzyme function and aerobic glycolysis in HIV-1 replication. Therefore, examining additional glycolytic enzymes under aerobic glycolytic conditions may provide insight into viral gene regulation.

Triosephosphate isomerase 1 (TPI1) is a key enzyme in glycolysis, which catalyzes the isomerization between dihydroxyacetone phosphate (DHAP) and glyceraldehyde-3-phosphate (GAP). As reviewed by Myers and Palladino, TPI1 also exhibits characteristics of a moonlighting protein (15). One intriguing aspect of its function is its capability to translocate to the nucleus and exhibit enzymatic activity. Zhang *et al.* reported that phosphorylated TPI1 translocates to the nucleus, where it converts its substrate DHAP into G3P, resulting in higher concentrations of acetate and enhanced histone acetylation, consequently leading to significant transcriptional alterations (16). Few studies have explored the relationship between TPI1 and viral infection; however, several reports suggest its involvement in viral infections in shrimp (17, 18). Although no direct link between TPI1 and human viral pathogens has been established, many viruses utilize host glycolytic pathways. Given that transcriptional activation is essential for efficient HIV-1 replication, investigating the involvement of TPI1 contributes to a more comprehensive understanding of HIV-1 replication.

In this study, we examined the relationship between TPI1 and HIV-1 replication, considering the metabolic state of cells. Under aerobic glycolytic conditions, a fraction of TPI1 translocates to the nucleus and promotes HIV-1 transcription. In non-HIV-1-infected PBMCs, nuclear TPI1 levels were unchanged regardless of metabolic state, suggesting that TPI1 nuclear translocation is observed in the context of HIV-1 infection. These findings identify a metabolic mechanism linking TPI1 function to HIV-1 transcription.

## Results

### Aerobic glycolytic conditions enhance HIV-1 transcription

**Figure 1:**
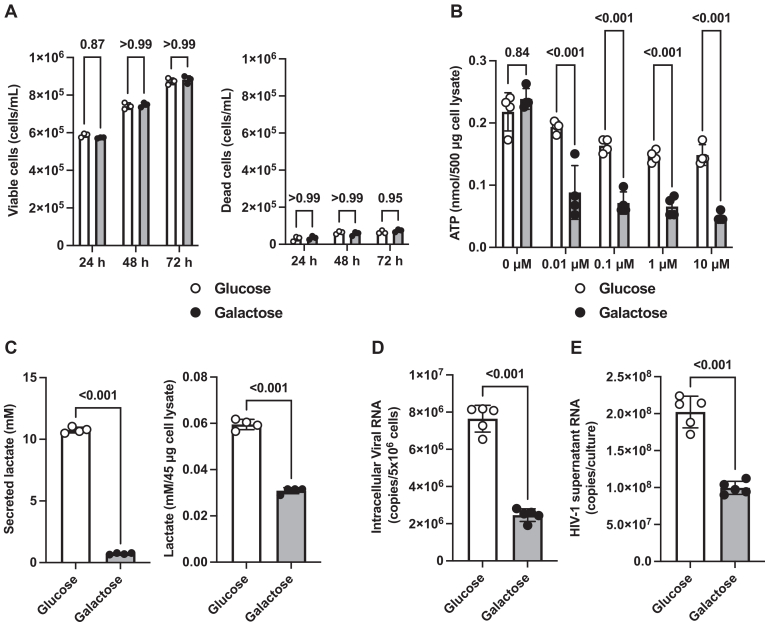
**Aerobic glycolytic conditions are associated with increased HIV-1 transcription.***A*, viability of CEM/LAV-1 cells grown in glucose- or galactose-containing medium. The number of viable (*left*) or dead (*right*) cells was determined by trypan blue staining. Bars indicate mean values, and error bars represent standard deviations from n = 3 independent biological replicates. *B*, intracellular ATP levels associated with CEM/LAV-1 cells cultured in glucose- or galactose-containing medium. The concentration indicates the final concentration of oligomycin. Bars indicate mean values, and error bars represent standard deviations from n = 4 independent biological replicates. *C*, amount of lactate associated with CEM/LAV-1 cells cultured in glucose- or galactose-containing medium. The *left* and *rightpanels* show the supernatant and intracellular lactate levels, respectively. Bars indicate mean values, and error bars represent standard deviations from n = 4 independent biological replicates. *D*, amount of viral RNA associated with CEM/LAV-1 cells grown in medium containing glucose or galactose. RNA was collected at 72 h after starting culture at 5 × 10^6^ cells. Bars indicate mean values, and error bars represent standard deviations from n = 5 independent biological replicates. *E*, amount of viral RNA associated with virus released into the culture supernatant from CEM/LAV-1 cells grown in medium containing glucose or galactose. Viruses were collected at 72 h after starting culture at 5 × 10^6^ cells in 10 ml. Bars indicate mean values, and error bars represent standard deviations from n = 5 independent biological replicates. For (*A* and *B*), statistical significance was assessed by two-way ANOVA with Bonferroni’s multiple comparisons test. For (*C*–*E*), statistical significance was assessedby an unpaired *t* test with Welch's correction.

To determine whether aerobic glycolysis influence HIV-1 gene expression, HIV-1-infected CEM/LAV-1 cells were cultured in glucose- or galactose-containing medium. Under our experimental conditions, the cell culture medium composition had no effect on cell proliferation or viability (Fig. 1*A*). In addition, ATP levels did not significantly differ between the two culture conditions in the absence of oligomycin (0 μM) (Fig. 1*B*). However, cells cultured in galactose-containing medium showed higher sensitivity to oligomycin, an inhibitor of OXPHOS (Fig. 1*B*). Furthermore, both secreted and intracellular lactate levels, an indicator of glycolytic flux, were lower in cells cultured in galactose-containing medium than in those cultured in glucose-containing medium (Fig. 1*C*). These results suggest that cells cultured in glucose-containing medium preferentially generate ATP through aerobic glycolysis, whereas those cultured in galactose-containing medium rely predominantly on OXPHOS for ATP production, as shown in our previous study (9). To assess the effect of metabolic conditions on HIV-1 transcription, we examined the transcriptional activity of the integrated HIV-1 genome. Cells cultured in galactose-containing medium showed decreased HIV-1 transcription (Fig. 1*D*) and virus release (Fig. 1*E*) relative to those cultured in glucose-containing medium. These findings suggest that aerobic glycolytic conditions facilitate HIV-1 transcription through metabolic-dependent regulatory mechanisms.

### Ser^80^-dependent regulation of TPI1 under aerobic glycolytic conditions enhances HIV-1 transcription

**Figure 2:**
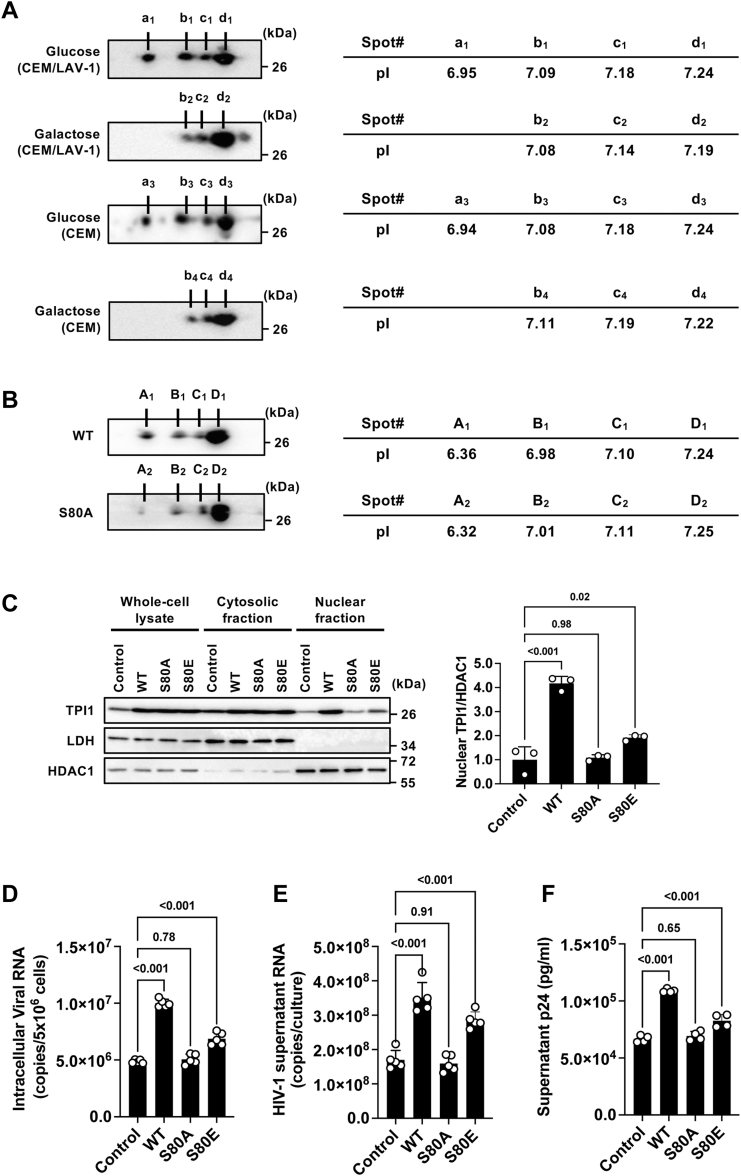
**Ser^80^ phosphorylation of TPI1 is associated with increased HIV-1 transcription.***A*, two-dimensional gel image of the lysate from CEM/LAV-1 or CEM cells grown in glucose- or galactose-containing medium, probed with an anti-TPI1 antibody. The pI values of TPI1 spots are shown to the *right* of each image. *B*, two-dimensional gel image of the lysate from CEM/LAV-1 cells transfected with WT or S80A TPI1, probed with an anti-TPI1 antibody. The pI values of TPI1 spots are shown to the *right* of each image. *C*, fractionation of CEM/LAV-1 cells transfected with WT, S80A, or S80E TPI1. LDH and HDAC1 were detected as the cytosolic and nuclear markers, respectively. Fractionation was performed using the same number of cells, and the amount of protein loaded in each lane was calculated by the BCA method. Quantification of western immunoblot band intensities is shown on the *right*. Nuclear TPI1 levels were normalized to HDAC1, and the average value of the control condition was set to 1. Bars indicate mean values, and error bars represent standard deviations from n = 3 independent biological replicates. *D*, amount of viral RNA associated with CEM/LAV-1 cells transfected with control, WT, S80A, or S80E TPI1. RNA was collected at 72 h after starting culture at 5 × 10^6^ cells. Bars indicate mean values, and error bars represent standard deviations from n = 5 independent biological replicates. *E*, amount of viral RNA associated with virus released into the culture supernatant from CEM/LAV-1 cells transfected with WT, S80A, or S80E TPI1. Viruses were collected at 72 h after starting culture at 5 × 10^6^ cells in 10 ml. Bars indicate mean values and error bars represent standard deviations from n = 5 independent biological replicates. *F*, amount of virus released into the culture supernatant from CEM/LAV-1 cells transfected with WT, S80A, or S80E TPI1 was measured on the basis of p24 antigen levels. Viruses were collected as described in (*E*). Bars indicate mean values and error bars represent standard deviations from n = 5 independent biological replicates. For (*C*–*F*), statistical significance was assessed by two-way ANOVA with Dunnett's multiple comparisons test.

Aerobic glycolysis conditions have been associated with the moonlighting roles of glycolytic enzymes that modulate HIV-1 gene expression (9). We therefore hypothesized that glycolytic enzymes may contribute to enhanced HIV-1 transcription under aerobic glycolytic conditions. Given that HIV-1 transcription occurs in the nucleus, we focused on glycolytic enzymes capable of nuclear translocation. Previous studies have shown that, in cancer cells, TPI1 undergoes phosphorylation at Ser^80^ and subsequently translocates to the nucleus (16). Cancer cells exhibit aerobic glycolysis, a phenomenon referred to as the Warburg effect. Based on these insights, we focused on TPI1. Protein phosphorylation typically shifts the isoelectric point (pI) toward a more acidic range. Therefore, we assessed the pI of TPI1 by two-dimensional electrophoresis. Four TPI1 isoforms with distinct pIs were detected in cells cultured in glucose-containing medium, whereas three isoforms were detected under galactose conditions. Notably, a distinct acidic isoform was observed specifically in cells cultured in glucose-containing medium (Fig. 2*A*). To test the involvement of Ser^80^, HIV-1-infected cells under aerobic glycolytic conditions were transfected with WT or the S80A TPI1 expression vector, and TPI1 pI profiles were analyzed. Overexpression of WT TPI1 increased the abundance of the acidic isoform and enhanced HIV-1 transcription, whereas the S80A mutant did not. The weak acidic signal observed in the S80A mutant likely reflects endogenous TPI1 (Fig. 2*B*). Together, these data are consistent with Ser80-dependent phosphorylation of TPI1 under aerobic glycolytic conditions in HIV-1-infected cells.

We next examined how Ser^80^ affects TPI1 nuclear localization and HIV-1 transcription. Subcellular fractionation showed that a fraction of TPI1 was present in the nucleus (Fig. 2*C*, control). Overexpression of WT TPI1 increased nuclear TPI1 levels (Fig. 2*C*, WT). In contrast, the phosphorylation-deficient S80A mutant did not increase nuclear TPI1 levels, whereas the phosphomimetic S80E mutant substantially increased nuclear accumulation (Fig. 2*C*, S80A and S80E). We then assessed HIV-1 transcription in infected cells (Fig. 2*D*). WT TPI1 overexpression increased HIV-1 transcription relative to control cells, whereas the S80A mutant did not. In addition, expression of the S80E mutant resulted in a significant increase in transcription levels relative to the control. Consistent with these changes, virus release into the culture supernatant—quantified by viral RNA and p24 measurements (Fig. 2, *E* and *F*)—paralleled nuclear TPI1 levels and HIV-1 transcription. Together, these findings suggest that Ser^80^-dependent phosphorylation of TPI1 under aerobic glycolytic conditions promotes nuclear translocation and enhances HIV-1 transcription.

### Nuclear TPI1 is associated with higher H3K27 acetylation and HIV-1 transcription levels

**Figure 3:**
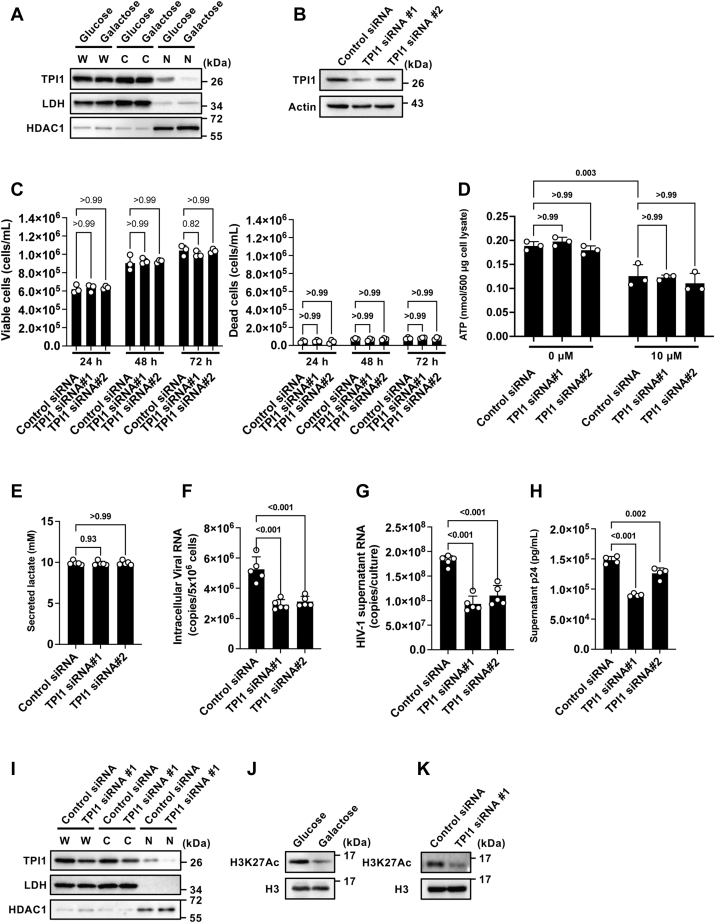
**Nuclear TPI1 levels correlate with H3K27 acetylation under distinct metabolic conditions.***A*, fractionation of CEM/LAV-1 cells grown in glucose- or galactose-containing medium. LDH and HDAC1 were detected as the cytosolic and nuclear markers, respectively. Fractionation was performed using an equal number of cells, and the amount of protein loaded in each lane was calculated by the BCA method. W, whole-cell lysate; *C*, cytosolic fraction; N, nuclear fraction. *B*, TPI1 siRNA knockdown efficiency was confirmed by western immunoblotting. TPI1 expression was analyzed in lysates from CEM/LAV-1 cells at 72 h after transfection with TPI1-specific or control siRNA. *C*, assessment of siRNA-induced cytotoxicity. Viable cells (*left*) and dead cells (*right*) were counted after trypan *blue* staining. Bars indicate mean values and error bars represent standard deviations from n = 3 independent biological replicates. *D*, intracellular ATP levels associated with siRNA-treated CEM/LAV-1 cells. Final concentrations of oligomycin used for treatment are shown at the *bottom*. Bars indicate mean values and error bars represent standard deviations from n = 3 independent biological replicates. *E*, secreted lactate levels in siRNA-transfected CEM/LAV-1 cells. Bars indicate mean values and error bars represent standard deviations from n = 5 independent biological replicates. *F*, amount of viral RNA associated with CEM/LAV-1 cells transfected with siRNA. RNA was collected at 72 h after starting culture at 5 × 10^6^ cells. Bars indicate mean values and error bars represent standard deviations from n = 5 independent biological replicates. *G*, amount of viral RNA associated with virus released into the culture supernatant from CEM/LAV-1 cells transfected with siRNA. Viruses were collected at 72 h after starting culture at 5 × 10^6^ cells in 10 ml. Bars indicate mean values and error bars represent standard deviations from n = 5 independent biological replicates. *H*, amount of virus released into the culture supernatant from CEM/LAV-1 cells transfected with siRNA was measured on the basis of p24 antigen levels. Viruses were collected as described in (*G*). Bars indicate mean values and error bars represent standard deviations from n = 5 independent biological replicates. *I*, fractionation of CEM/LAV-1 cells transfected with siRNA. LDH and HDAC1 were detected as the cytosolic and nuclear markers, respectively. Fractionation was performed using the same number of cells, and the amount of protein loaded in each lane was calculated by the BCA method. C, cytosolic fraction; N, nuclear fraction; W, whole cell lysate. *J*, detection of histone acetylation using lysates from cells grown in glucose- or galactose-containing medium. Ac, acetylation. *K*, detection of histone acetylation using lysates from cells transfected with siRNA. Ac, acetylation. For (*C* and *D*), statistical significance was assessed by two-way ANOVA with Bonferroni’s multiple comparisons test. For (*E*–*H*), statistical significance was assessed by one-way ANOVA with Dunnett's multiple comparisons test.

We next examined whether reduced nuclear TPI1 contributes to the lower HIV-1 transcription observed under galactose conditions. Subcellular fractionation showed that total TPI1 levels were comparable between glucose- and galactose-grown HIV-1-infected cells (Fig. 3*A*, whole-cell lysate fractions). In contrast, nuclear TPI1 levels were markedly reduced under galactose conditions (Fig. 3*A*, nuclear fractions). Based on these findings, we performed TPI1 knockdown under aerobic glycolytic conditions (Fig. 3*B*). TPI1 knockdown did not affect cell viability or basal ATP levels (Fig. 3, *C* and *D*). Treatment with 10 μM oligomycin reduced ATP levels, consistent with an OXPHOS contribution to ATP production. Importantly, this response was preserved upon TPI1 knockdown, suggesting that the balance between glycolysis and OXPHOS was not grossly altered (Fig. 3*D*). In addition, the amount of secreted lactate derived from glycolysis remained unchanged (Fig. 3*E*). Together, these results indicate that TPI1 was reduced without overtly altering the measured metabolic readouts under our experimental conditions. TPI1 knockdown reduced HIV-1 transcription (Fig. 3*F*). It also reduced viral genomic RNA and p24 antigen levels in the culture supernatant (Fig. 3, *G* and *H*). Consistently, TPI1 knockdown reduced nuclear TPI1 levels (Fig. 3*I*). Because chromatin state influences HIV-1 transcription, we examined histone H3K27 acetylation. H3K27 acetylation, a marker of transcriptionally active chromatin, was reduced under galactose conditions (Fig. 3*J*). Similarly, TPI1 knockdown decreased H3K27 acetylation levels (Fig. 3*K*). Together, these results suggest that nuclear TPI1 is associated with higher H3K27 acetylation levels and enhanced HIV-1 transcription under aerobic glycolytic conditions.

### TPI1 nuclear translocation is observed under aerobic glycolytic conditions in HIV-1-infected cells

**Figure 4:**
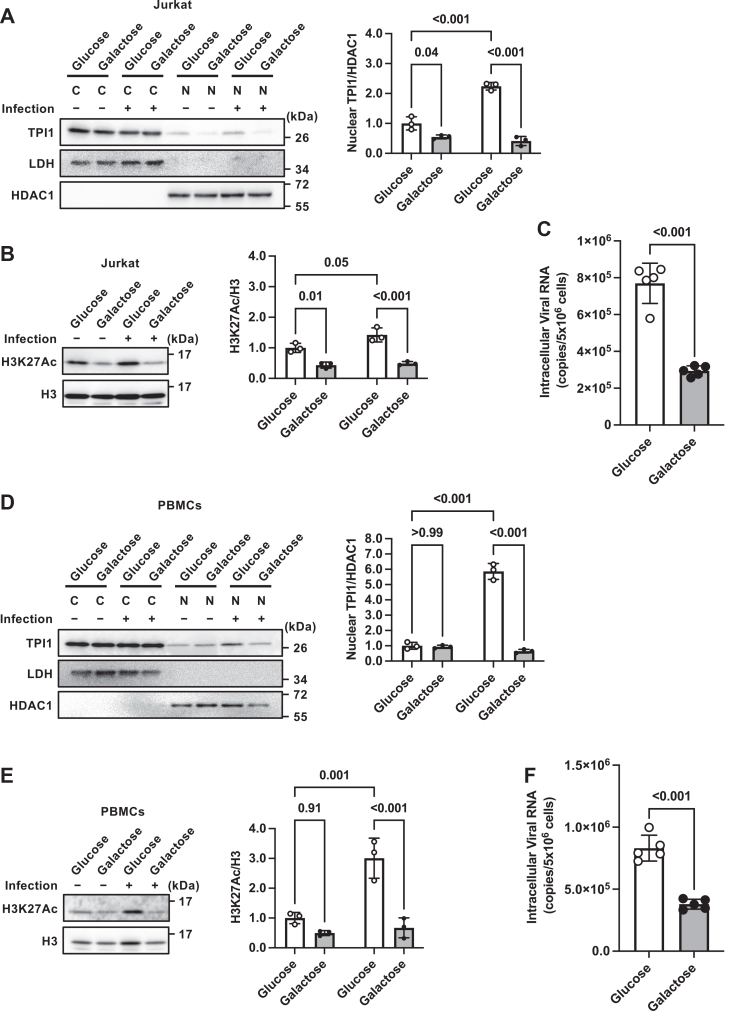
**Nuclear TPI1 localization in HIV-1–infected Jurkat cells and PBMCs under distinct metabolic conditions.***A*, fractionation of uninfected or HIV-1-infected Jurkat cells cultured in glucose- or galactose-containing medium for 72 h. *C*, cytosolic fraction; N, nuclear fraction. Infection status is indicated as “−” (uninfected) and “+” (HIV-1-infected). Quantification of western immunoblot band intensities is shown on the *right*. Nuclear TPI1 levels were normalized to HDAC1, and the average value of the glucose condition was set to 1. Bars indicate mean values, and error bars represent standard deviations from n = 3 independent biological replicates. *B*, detection of histone acetylation in lysates from Jurkat cells. Quantification of western immunoblot band intensities is shown on the *right*. Acetylated H3K27 levels were normalized to H3, and the average value of the glucose condition was set to 1. Bars indicate mean values, and error bars represent standard deviations from n = 3 independent biological replicates. Ac, acetylation. *C*, amount of viral RNA associated with HIV-1-infected Jurkat cells grown in medium containing glucose or galactose. RNA was collected at 72 h after starting culture at 5 × 10^6^ cells in 10 ml. Bars indicate mean values and error bars represent standard deviations from n = 5 independent biological replicates. *D*, fractionation of uninfected or HIV-1-infected PBMCs cultured in glucose- or galactose-containing medium for 72 h. *C*, cytosolic fraction; N, nuclear fraction. Infection status is indicated as “−” (uninfected) and “+” (HIV-1-infected). Quantification of western immunoblot band intensities is shown on the *right*. Nuclear TPI1 levels were normalized to HDAC1, and the average value of the glucose condition was set to 1. Bars indicate mean values, and error bars represent standard deviations from n = 3 independent biological replicates. *E*, detection of histone acetylation in lysates from PBMCs. Cells were either non- or HIV-1-infected and cultured in glucose- or galactose-containing media. Quantification of western immunoblot band intensities is shown on the *right*. Acetylated H3K27 levels were normalized to H3, and the average value of the glucose condition was set to 1. Bars indicate mean values, and error bars represent standard deviations from n = 3 independent biological replicates. Ac, acetylation. *F*, amount of viral RNA associated with HIV-1-infected PBMCs grown in medium containing glucose or galactose. RNA was collected at 72 h after starting culture at 5 × 10^6^ cells in 10 ml. Bars indicate mean values and error bars represent standard deviations from n = 5 independent biological replicates. For (*A*, *B*, *D* and *E*), statistical significance was assessed by two-way ANOVA with Bonferroni’s multiple comparisons test. For (*C* and *F*), statistical significance was assessed by an unpaired *t* test with Welch's correction.

In the preceding experiments, we primarily used chronically HIV-1-infected cell lines. We next asked whether TPI1 nuclear translocation is also observed in the setting of acute HIV-1 infection. First, we analyzed the localization of TPI1 in Jurkat cells. In uninfected Jurkat cells, nuclear TPI1 levels were reduced under galactose conditions (Fig. 4*A*). In HIV-1-infected Jurkat cells, galactose culture was associated with reduced nuclear TPI1 levels (Fig. 4*B*), accompanied by decreased H3K27 acetylation (Fig. 4*C*) and lower HIV-1 transcription (Fig. 4*D*). We then extended the analysis to PBMCs from healthy donors. In uninfected PBMCs, nuclear TPI1 levels were similar between glucose and galactose conditions (Fig. 4*E*). In contrast, in HIV-1-infected PBMCs, nuclear TPI1 levels were reduced under galactose conditions (Fig. 4*F*). This was accompanied by decreased H3K27 acetylation and reduced HIV-1 transcription (Fig. 4, *G* and *H*). While many cancer cells exhibit aerobic glycolysis (the Warburg effect), HIV-1 infection has been reported to increase glycolytic flux in PBMCs (8). Together, these results suggest that aerobic glycolytic conditions are associated with TPI1 nuclear translocation in HIV-1–infected cells and link cellular metabolic state to HIV-1 transcription.

## Discussion

Viruses depend on host cellular machinery, and host's physiological state can strongly influence viral gene expression. Previous studies have reported that HIV-1 infection is associated with increased glycolytic flux, which supports viral gene expression (8, 9). Here, we identify Ser^80^-dependent regulation of TPI1 as one mechanism linking metabolic state to HIV-1 transcription. These findings underscore the importance of understanding how metabolic state modulates protein function to influence HIV-1 transcription in immune cells.

We observed that under aerobic glycolysis conditions in HIV-1-infected cells, TPI1 accumulates in the nucleus and is associated with increased H3K27 acetylation. Previous studies have shown that HIV-1 transcription is epigenetically regulated, and histone acetylation enhances transcriptional activity (19, 20, 21). Integrated HIV-1 DNA preferentially localizes to euchromatic regions (22). These observations raise the possibility that nuclear TPI1 may contribute to a chromatin environment permissive for HIV-1 transcription. However, the mechanism underlying the nuclear import of phosphorylated TPI1 remains to be elucidated. TPI1 does not contain a canonical nuclear localization signal (NLS), and its phosphorylation is not predicted to generate one. Interestingly, the glycolytic enzyme PKM2 contains a Ser-Pro motif that is phosphorylated by ERK2, and phosphorylation at Ser^37^ promotes Pin1-dependent conformational changes that enable interaction with importin-α5 and nuclear translocation (23). The phosphorylation site of TPI1 identified by Zhang *et al.* is a Ser-Pro motif (Ser^80^-Pro^81^) (16), suggesting that CDK2-phosphorylated TPI1 may be recognized by Pin1. Because Pin1 contains an NLS, interaction between phosphorylated TPI1 and Pin1 may facilitate TPI1 nuclear import. As proposed for PKM2, Pin1-mediated conformational changes in TPI1 could enhance interactions with nuclear transport factors such as importin-α5. Alternatively, TPI1 may enter the nucleus through Pin1-and importin-α5–independent mechanisms, potentially influencing transcriptional regulation under distinct cellular conditions. Further investigation into the interplay among glucose metabolism, kinase activity, and nuclear transport may clarify how metabolic state confers functional diversity on proteins and influences epigenetic regulation.

TPI1 has attracted considerable attention in cancer research owing to its diverse roles in tumor progression. Activation of the PI3K/AKT/mTOR signaling pathway associated with TPI1 promotes protein synthesis, cell proliferation, and tumor-promoting processes such as epithelial-mesenchymal transition (24). In breast cancer cells, TPI1 overexpression increases mTOR phosphorylation, concomitant with increased glycolytic activity and metastatic potential (25). The expression and function of TPI1 are context-dependent: it promotes tumor progression in breast, pancreatic, and lung cancers, whereas tumor-suppressive roles have been reported in hepatocellular carcinoma (24, 25, 26, 27, 28). These observations highlight the context-dependent roles of TPI1 and suggest that targeting cancer-specific metabolic dependencies may inform therapeutic strategies. In this study, nuclear TPI1 levels were not elevated in uninfected PBMCs but were increased in HIV-1-infected PBMCs under aerobic glycolytic conditions. This contrast underscores the importance of defining the structural determinants and interaction networks that govern TPI1 function in distinct cellular contexts, including cancer and HIV-1 infection, and may inform future antiviral strategies.

A state contrasting active HIV-1 transcription is latency. Latent HIV-1 infection is characterized by replication-competent but transcriptionally silenced proviruses (29). HIV-1 latency can arise through multiple mechanisms that silence viral gene expression, including epigenetic DNA modifications (30, 31). We found that inhibition of glycolysis by replacing glucose with galactose reduced viral production. Shytaj *et al.* reported that enhanced glycolysis during the early phase of HIV-1 infection supports productive replication, whereas gradual downregulation of glycolysis later contributes to the establishment and maintenance of latency (32). Furthermore, Mutascio *et al.* reported that CD8^+^ T cells contribute to the metabolic reprogramming of CD4^+^ T cells, leading to the suppression of glycolysis and induction of latency (33). Together, these studies indicate that the cellular metabolic state profoundly influences HIV-1 transcriptional activity and infection outcome. It will be important to determine whether TPI1 regulation is linked to the establishment or maintenance of latency. If TPI1 localization or post-translational modification contributes to latency, these features may serve as candidate markers of latently infected cells. Future studies should examine TPI1 regulation under conditions of suppressed HIV-1 transcription, including latency. Because latency remains a major barrier to HIV-1 eradication, understanding mechanisms that control HIV-1 transcription is crucial. The present findings suggest that metabolic regulation of TPI1 may represent a potential avenue for therapeutic intervention.

## Experimental procedures

### Cells

A chronically HIV-1_LAV-1_-infected T-cell line (CEM/LAV-1 cells), Jurkat cells or PBMCs were cultured at 37 °C in 5% CO_2_, as previously described (34). PBMCs were purchased from Precision for Medicine (samples were de-identified and collected with informed consent under protocols approved by the appropriate institutional review boards) and stimulated with Dynabeads Human T-Activator CD3/CD28 (Thermo Fisher Scientific) in the presence of 30 IU/ml IL-2 (Shionogi). Glucose- or galactose-containing medium was prepared by supplementing glucose-free basal medium with 10 mM glucose or 10 mM galactose. Cell viability was assessed by trypan blue exclusion. ATP and lactate levels were measured using the ATP Colorimetric/Fluorometric Assay Kit (Abcam) and Lactate Assay Kit-WST (Dojindo), respectively, according to the manufacturers’ instructions. For inhibition of OXPHOS, cells were treated with oligomycin (Selleck Chemicals) prior to ATP measurement. In addition, experimental conditions were based on those described previously (9). To prepare infected Jurkat cells and PBMCs, Jurkat cells and PBMCs were infected *via* spinoculation (1200*g* for 2 h at 24 °C) with 100 ng of the p24 antigen of HIV-1_LAV−1_ per 1.0 × 10^6^ cells. Infection efficiency was confirmed by RT-qPCR quantification of HIV-1 RNA. This study was conducted in accordance with the ethical principles of the Declaration of Helsinki.

### HIV-1 genome detection

Intracellular viral RNA was collected using the RNeasy Mini Kit (QIAGEN) in accordance with the manufacturer’s instructions. Intracellular viral RNA was quantified by RT-qPCR using the One Step PrimeScript III RT-qPCR Mix (Takara Bio) with the following primers and probe: forward primer MH531 (5′-TGTGTGCCCGTCTGTTGTGT-3′), reverse primer MH532 (5′-GAGTCCTGCGTCGAGAGAGC-3′), and probe LRT-P (5′-(FAM)-CAGTGGCGCCCGAACAGGGA-(TAMRA)-3′) (35). Viral RNA in culture supernatants was extracted using the QIAamp Viral RNA Mini Kit (QIAGEN) in accordance with the manufacturer’s instructions, and then reverse-transcribed using the SuperScript VILO cDNA Synthesis Kit (Thermo Fisher Scientific). Synthesized cDNA was quantified by qPCR using the same primer-probe set described above.

### Protein detection

Two-dimensional electrophoresis was performed as previously described (11). Briefly, cell lysates were first separated according to their isoelectric points within a pH range of 3 to 10, followed by molecular weight-dependent separation by SDS-PAGE. Subcellular fractionation was performed using the Subcellular Protein Fractionation Kit for Cultured Cells (Thermo Fisher Scientific) according to the manufacturers’ instructions. In Western immunoblotting, proteins were transferred on PVDF membranes, and chemiluminescent signals (SuperSignal West Pico PLUS Chemiluminescent Substrate, Thermo Fisher Scientific) were detected using Fusion Solo 7S Edge (Vilber Bio Imaging). Primary antibodies used in this study were commercially available and validated by the manufacturers: anti-TPI1 antibody (Proteintech), anti-actin antibody (FUJIFILM Wako Pure Chemical Corporation), anti-LDH antibody (Abcam), anti-HDAC1 antibody (Cell Signaling Technology), anti-histone H3 antibody (Abcam), anti-histone H3K27ac antibody (Abcam), and anti-p24 antibody (ViroGen). Signal intensity was measured using Image J 1.54*g* Software.

### Transfection

Transfections were performed using the Neon transfection system (Thermo Fisher Scientific) in accordance with the manufacturer’s instructions, with the following parameters; 1500 V, 10 ms, and 3 pulses. For TPI1 knockdown, cells were transfected with commercially available and validated Silencer TPI1 siRNA at 200 nM (siRNA ID, #1: s14339, #2: s224747; Thermo Fisher Scientific). For TPI overexpression or S80A and S80E expression, the coding region of human TPI1 or its mutants was cloned into pEBMulti-Neo (Wako Pure Chemical Industries). S80A and S80E were verified by sequencing. As a control, cells were transfected with the same vector lacking the TPI1 coding sequence.

### Statistical analyses

Statistical analyses were performed using GraphPad Prism (version 10). Details of the statistical tests used are provided in the figure legends. Individual data points represent independent biological replicates.

## Data availability

All data are included in the manuscript and are available from the corresponding author upon reasonable request.

## Conflict of interest

The authors declare that they have no conflicts of interest with the contents of this article.
